# Supplementary material for: Pooled multicolour tagging for visualizing subcellular protein dynamics
Source: Nat Cell Biol. 2024 Apr 19;26(5):745–56. doi: 10.1038/s41556-024-01407-w (PMC11098740; doi:10.1038/s41556-024-01407-w)

nature cell biology

Article

https://doi.org/10.1038/s41556-024-01407-w

Pooled multicolour tagging for visualizing subcellular protein dynamics

In the format provided by the authors and unedited

Supplementary Figure 1. FACS gating strategy. The forward and sideward scatters are used to select the starting cell populations. Single cells are then selected using FSC-A and FSC-H and cells are quantified and/or sorted based on their green or red fluorescence in the FITC and PI channels, respectively. Only the panels with the FITC and PI intensities are shown in the Extended Data Figures of the manuscript. Panel B of the example shown here is part of Extended Data Figure 1d.

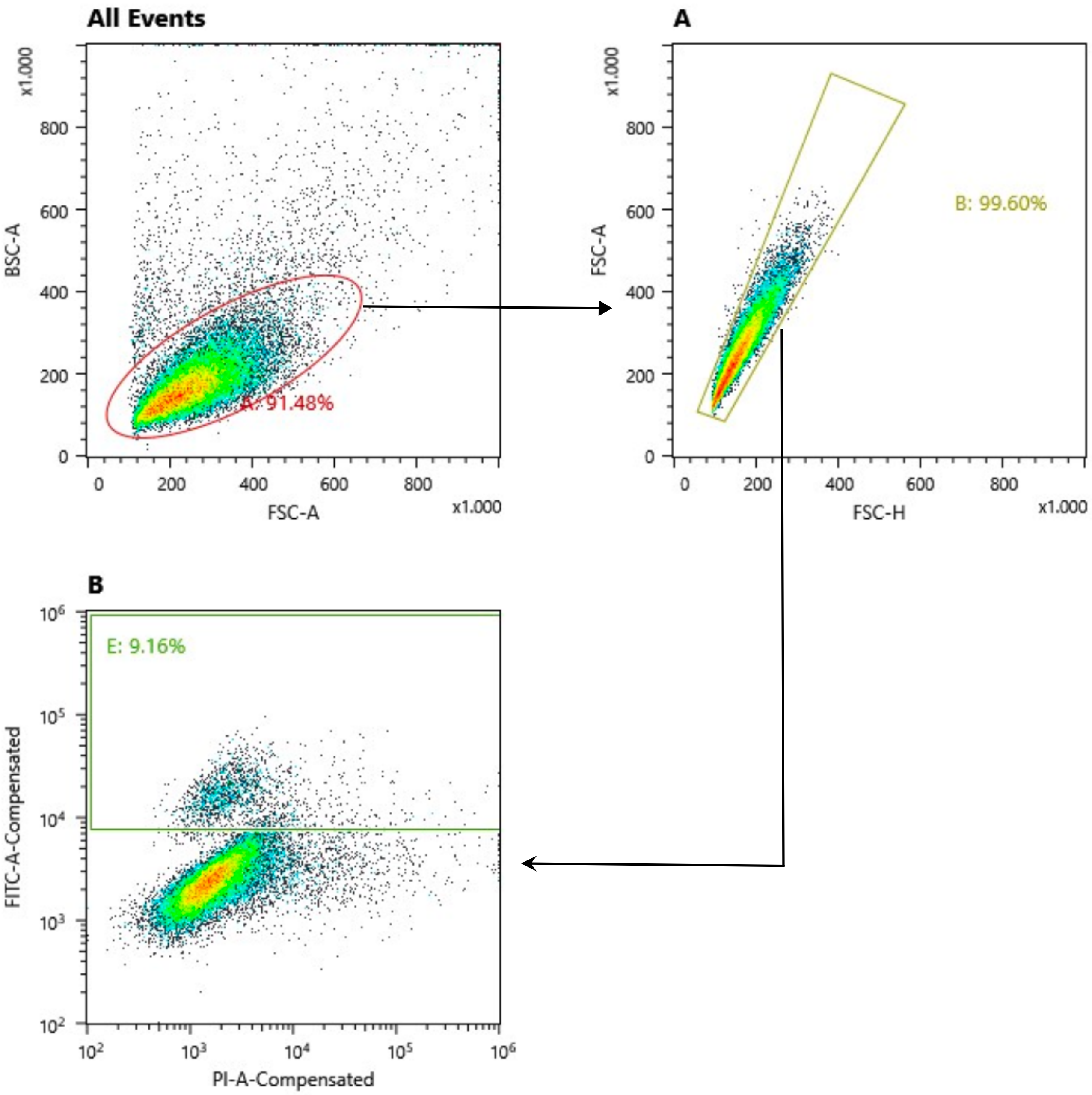

Supplementary Figure 2. Additional examples of segmentation. a-d, Segmentation for 4 representative fields of view. Tagged channels: overlay of GFP (green), mScarlet (red), BFP (blue). Structural channels: overlay of membrane marker mAmetrine (yellow) and nuclear marker miRFP (red). Scale bars: 50 μm. **e**, Representative example segmentation of mitotic cells. White arrows point to cells undergoing mitosis, the large majority of which are discarded during filtering. Scale bars: 50 μm.

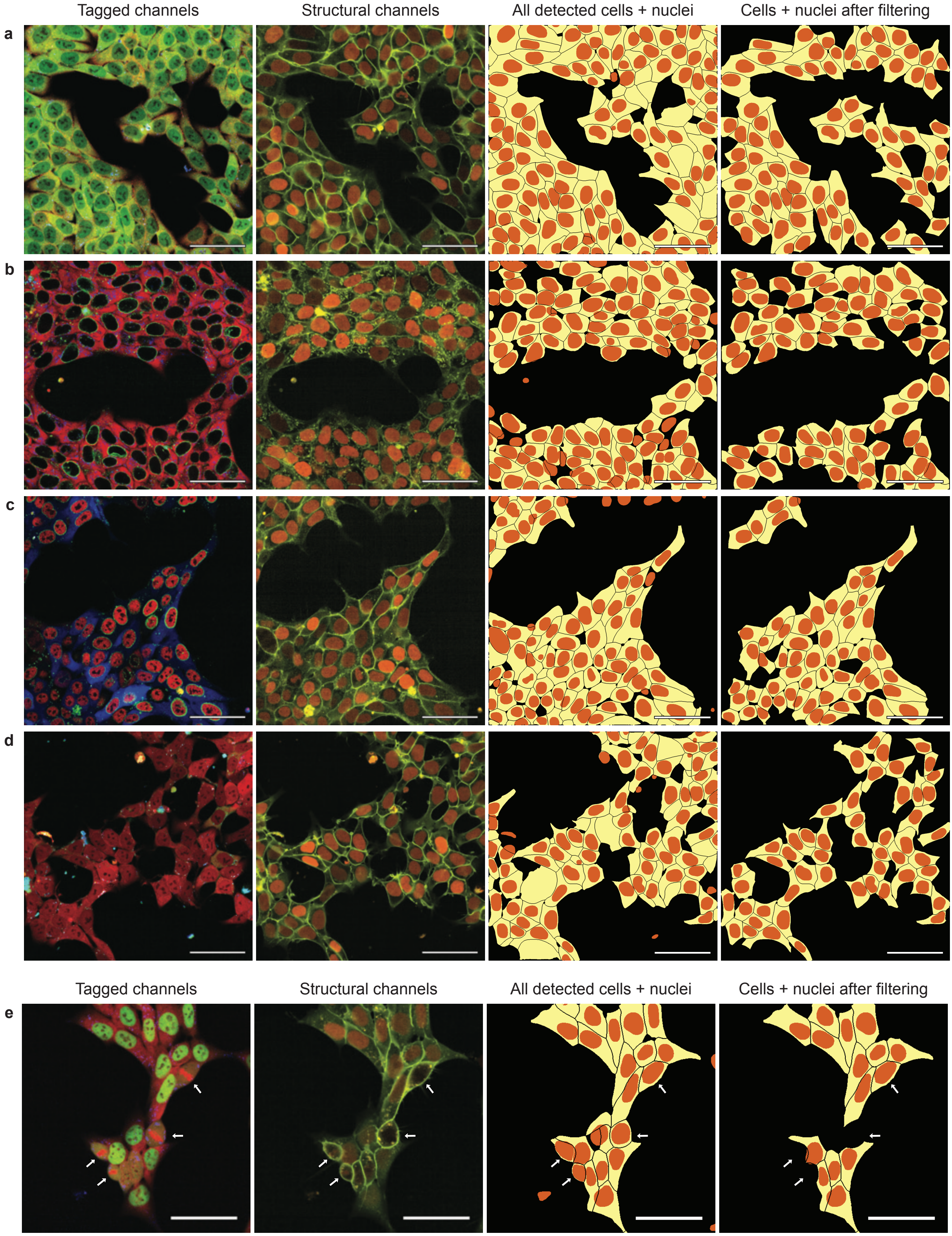

Supplement: Supplementary file 1 — Supplementary Figs. 1 and 2. [file 41556_2024_1407_MOESM1_ESM.pdf]
