# Supplementary material for: Pooled multicolour tagging for visualizing subcellular protein dynamics
Source: Nat Cell Biol. 2024 Apr 19;26(5):745–56. doi: 10.1038/s41556-024-01407-w (PMC11098740; doi:10.1038/s41556-024-01407-w)
Supplement: Supplementary file 2 — Reporting Summary [file 41556_2024_1407_MOESM2_ESM.pdf]

nature portfolio

Corresponding author(s): Stefan Kubicek

Last updated by author(s): Mar 8, 2024

Reporting Summary

Nature Portfolio wishes to improve the reproducibility of the work that we publish. This form provides structure for consistency and transparency in reporting. For further information on Nature Portfolio policies, see our Editorial Policies and the Editorial Policy Checklist.

Statistics

For all statistical analyses, confirm that the following items are present in the figure legend, table legend, main text, or Methods section.

n/a	Confirmed	
☐	☒	The exact sample size (n) for each experimental group/condition, given as a discrete number and unit of measurement
☐	☒	A statement on whether measurements were taken from distinct samples or whether the same sample was measured repeatedly
☐	☒	The statistical test(s) used AND whether they are one- or two-sided<br>*Only common tests should be described solely by name; describe more complex techniques in the Methods section.*
☒	☐	A description of all covariates tested
☐	☒	A description of any assumptions or corrections, such as tests of normality and adjustment for multiple comparisons
☐	☒	A full description of the statistical parameters including central tendency (e.g. means) or other basic estimates (e.g. regression coefficient) AND variation (e.g. standard deviation) or associated estimates of uncertainty (e.g. confidence intervals)
☐	☒	For null hypothesis testing, the test statistic (e.g. F, t, r) with confidence intervals, effect sizes, degrees of freedom and P value noted<br>*Give P values as exact values whenever suitable.*
☒	☐	For Bayesian analysis, information on the choice of priors and Markov chain Monte Carlo settings
☒	☐	For hierarchical and complex designs, identification of the appropriate level for tests and full reporting of outcomes
☒	☐	Estimates of effect sizes (e.g. Cohen's d, Pearson's r), indicating how they were calculated

Our web collection on statistics for biologists contains articles on many of the points above.

Software and code

Policy information about availability of computer code

Data collection	Harmony High-Content Imaging and Analysis Software, version 6 (Perkin Elmer), Sony Cell Sorter Software 2.1.6, GuideScan sgRNA database version 1.0
Data analysis	python 3.9.15, slurm 21.08.8, pandas 1.3.0, matplotlib-base 3.6.2, numpy 1.23.5, seaborn 0.11.2, cellpose 0.6.1, nucleAIzer-backend 0.2.1, scikit-image 0.19.1, CellProfiler 4.2.1, scikit-learn 1.1.3, umap 0.1.1, scipy 1.9.3, opencv 4.7.0, Prism 9.0<br><br>The image processing pipeline and hit calling code is deposited at https://github.com/reinisj/intron_tagging. DOI: 10.5281/zenodo.10598625

For manuscripts utilizing custom algorithms or software that are central to the research but not yet described in published literature, software must be made available to editors and reviewers. We strongly encourage code deposition in a community repository (e.g. GitHub). See the Nature Portfolio guidelines for submitting code & software for further information.

Data

Policy information about availability of data

All manuscripts must include a data availability statement. This statement should provide the following information, where applicable:

- Accession codes, unique identifiers, or web links for publicly available datasets
- A description of any restrictions on data availability
- For clinical datasets or third party data, please ensure that the statement adheres to our policy

All data supporting the findings of this study are available within the article its Supplementary Information and the online resource vpcells.cemm.at.

Research involving human participants, their data, or biological material

Policy information about studies with human participants or human data. See also policy information about sex, gender (identity/presentation), and sexual orientation and race, ethnicity and racism.

Reporting on sex and gender	n/a
Reporting on race, ethnicity, or other socially relevant groupings	n/a
Population characteristics	n/a
Recruitment	n/a
Ethics oversight	n/a

Note that full information on the approval of the study protocol must also be provided in the manuscript.

Field-specific reporting

Please select the one below that is the best fit for your research. If you are not sure, read the appropriate sections before making your selection.

☒ Life sciences ☐ Behavioural & social sciences ☐ Ecological, evolutionary & environmental sciences

For a reference copy of the document with all sections, see nature.com/documents/nr-reporting-summary-flat.pdf

Life sciences study design

All studies must disclose on these points even when the disclosure is negative.

Sample size	No statistical methods were used to pre-determine sample sizes but our sample sizes are similar to those reported in previous publications (10, 18). Sample size was chosen to balance replication and efficiency in the experiments. The exact sample size of the associated experiments are described in the Method and Figure legends. There was in general good correlation between replicates for the different experiments justifying the chosen sample size.
Data exclusions	No data were excluded.
Replication	All experiments that were not performed in a pooled format, were successfully replicated in at least two biologically independent replicates as indicated in the figures and figure legends. The pooled protein tagging experiments using the genome-wide and focused sgRNA libraries were performed in multiple cell lines as indicated and as a single replicate per cell line. Conclusions derived from these pooled protein tagging experiments, including predicted sgRNA efficiencies were validated using individual sgRNAs in an arrayed format in experiments that were replicated three times as indicated in the corresponding figures. The pooled drug screen was performed in a single replicate. Validation experiments to confirm phenotypes observed in the pooled drug screen were successfully replicated in at least two biologically independent replicates, their numbers are indicated in the corresponding figures or their legend.
Randomization	All experiments were performed with molecular biological techniques. No randomization, but independent replicates were performed.
Blinding	Blinding was not used in this study.

Reporting for specific materials, systems and methods

We require information from authors about some types of materials, experimental systems and methods used in many studies. Here, indicate whether each material, system or method listed is relevant to your study. If you are not sure if a list item applies to your research, read the appropriate section before selecting a response.

Materials & experimental systems

n/a	Involved in the study
☐	☒ Antibodies
☐	☒ Eukaryotic cell lines
☒	☐ Palaeontology and archaeology
☒	☐ Animals and other organisms
☒	☐ Clinical data
☒	☐ Dual use research of concern
☒	☐ Plants

Methods

n/a	Involved in the study
☒	☐ ChIP-seq
☐	☒ Flow cytometry
☒	☐ MRI-based neuroimaging

Antibodies

Antibodies used	XPO1 (CRM1) polyclonal antibody: Novus Biologicals, NB100-79802<br>β-actin monoclonal antibody: Abcam, ab8224, clone number: mAbcam 8224
Validation	XPO1 (CRM1) polyclonal antibody: Validated using other independent antibodies and with different biological strategies (https://www.novusbio.com/products/crm1-antibody_nb100-79802#datasheet)<br>β-actin monoclonal antibody: Frequently used loading control antibody with >470 references (https://www.abcam.com/products/primary-antibodies/beta-actin-antibody-mabcam-8224-loading-control-ab8224.html)

Eukaryotic cell lines

Policy information about cell lines and Sex and Gender in Research

Cell line source(s)	HAP1: Haplogen (now Horizon Discovery, C631), HEK293T: ATCC CRL-3216
Authentication	Cell lines were not authenticated.
Mycoplasma contamination	Cell lines were tested every two months for mycoplasma contamination using PCR - with negative results.
Commonly misidentified lines (See ICLAC register)	No commonly misidentified cell lines were used in this study.

Flow Cytometry

Plots

Confirm that:

☒ The axis labels state the marker and fluorochrome used (e.g. CD4-FITC).

☒ The axis scales are clearly visible. Include numbers along axes only for bottom left plot of group (a 'group' is an analysis of identical markers).

☒ All plots are contour plots with outliers or pseudocolor plots.

☒ A numerical value for number of cells or percentage (with statistics) is provided.

Methodology

Sample preparation	Adherent HAP1 or HEK293T cells were detached using trypsin and centrifuged (280 g, 5 min). Cell pellets were resuspended in PBS with 5% FBS before being transferred to polystyrene round bottom tubes with a cell strainer cap.
Instrument	Sony SH800S, model type: LE-SH800SZDCPL
Software	Sony Cell Sorter Software
Cell population abundance	Purity of post-sort fractions were analyzed by flow cytometry and live-cell imaging.
Gating strategy	The forward and sideward scatter was used to select the starting cell populations. Single cells were then selected using FSC-A and FSC-H and finally, cells were selected based on their green or red fluorescence as shown in the figures of the manuscript.

☒ Tick this box to confirm that a figure exemplifying the gating strategy is provided in the Supplementary Information.
