# Supplementary material for: Pooled multicolour tagging for visualizing subcellular protein dynamics
Source: Nat Cell Biol. 2024 Apr 19;26(5):745–56. doi: 10.1038/s41556-024-01407-w (PMC11098740; doi:10.1038/s41556-024-01407-w)

Unprocessed blots of Extended Data Fig. 9: The dotted boxes indicate the sections shown in the figure.

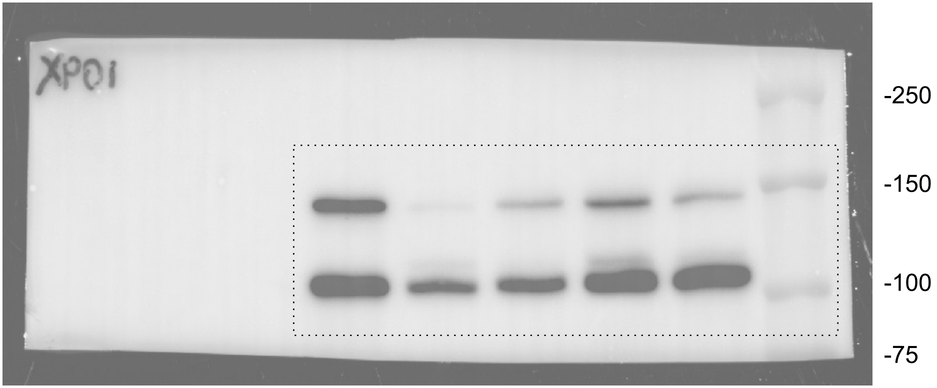

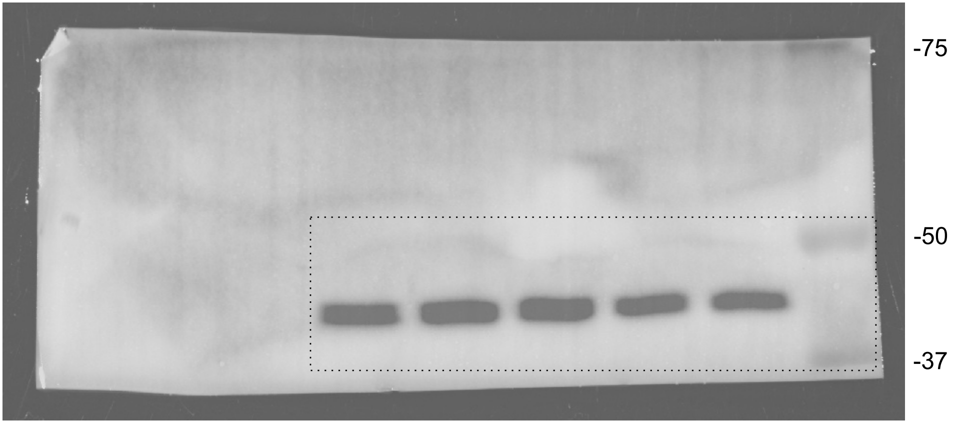

Supplement: Supplementary file 14 — Source Data Extended Data Fig. 9. [file 41556_2024_1407_MOESM14_ESM.pdf]
